# Comparison of severity of immunized versus non-immunized COVID-19 patients admitted to ICU: A prospective observational study

**DOI:** 10.1016/j.amsu.2021.102951

**Published:** 2021-10-15

**Authors:** Huda Mhawish, Ahmed Mady, Faisal Alaklobi, Waleed Aletreby, Tasmiya Asad, Mohammed Alodat, Abdulrahman Alharthy, Basheer Abdulrahman, Saleh Almahwi, Ziad A. Memish

**Affiliations:** a Critical Care Department, King Saud Medical City, Riyadh, Saudi Arabia; b Anesthesia Department, Faculty of Medicine, Tanta University, Tanta, Egypt; c Pediatrics Hospital, King Saud Medical City, Riyadh, Saudi Arabia; d Research & Innovation Centre King Saud Medical City, Riyadh, Saudi Arabia; e College of Medicine, Alfaisal University, Riyadh, Saudi Arabia; f Hubert Department of Global Health, Rollins School of Public Health, Emory University, Atlanta, GA, USA

**Keywords:** COVID-19, Immunization, Severity, ICU

## Abstract

**Background:**

Vaccines against COVID-19 show high efficacy, yet, infection is still being detected among immunized patients, although with blunted severity. The purpose of this study was to assess the severity of COVID-19 infection among immunized versus non-immunized COVID-19 patients admitted to ICU.

**Method:**

A prospective observational cohort study, including all COVID-19 patients admitted to intensive care unit between January 1st, 2021 and June 30th, 2021 were eligible for inclusion. A comparison of severity upon hospitalization of immunized versus non-immunized patients on a 7-level ordinal scale was conducted, using ordinal logistic regression.

**Results:**

592 patients were enrolled, 524 (88.5%) non-immunized, 63 (10.6%) partially immunized, and 5 (0.9%) fully immunized, partially and fully immunized patients were grouped together. Majority of immunized patients (86.7%) were symptomatic before 21 days of immunization. Non-immunized group had fewer patients in the lower severity categories, while more patients in the higher severity categories compared to immunized group. At least one dose of immunization was associated with reduction of odds of moving up severity scale (OR = 0.2 [95% CI: 0.15–0.4]; p < 0.001) in a well fitted ordinal logistic regression model. At least one dose of immunization was associated with lower adjusted odds of 30 day all-cause mortality (OR = 0.45 [95% CI: 0.23–0.89]; p = 0.02). Non-immunized group had higher mortality rate (43.9% versus 29.4% [95% CI: 1.5 to 25.8]; p = 0.02).

**Conclusion:**

Most COVID-19 patients admitted to ICU were non-immunized, most of the partially immunized patients got infected before immunity could develop, and fully immunized patients were likely non-responders. At least one dose of immunization significantly decreases severity of the disease across all ordinal severity categories, and is significantly associated with lower 30 day all-cause mortality. Accordingly, immunization status may have to be considered when deciding on disposition of COVID-19 patients at the point of triage.

## Highlights

• Vaccines against COVID-19 show high efficacy in preventing hospitalization and death.
• In our report most COVID-19 patients admitted to ICU were non-immunized.
• At least one dose of immunization significantly decreases severity of the disease.
• Partially immunized patients get infected before immunity could develop.
• Fully immunized patients were likely non-responders.

## 1 Introduction

Since the beginning of corona virus disease 2019 (COVID-19) infections, and its declaration as a pandemic on March 11, 2020 [1], the total number exceeded 200 million cases and the death toll has exceeded four million worldwide, as of August 13, 2021 [2]. Various strategies and efforts have been put forward to combat the pandemic, one of the most effective of which is the mass vaccination of the largest possible portion of population, particularly those at high risk [3]. Accordingly, an against-the-clock endeavor began to create such a vaccine, that ultimately resulted in the authorization of several vaccines in Europe and the United States, based on phase 2/3 safety and efficacy randomized clinical trials (RCT), reporting high efficacy [[4], [5], [6]].

Despite providing a high degree of protection against infection, no vaccine is 100% effective, it follows; that some vaccinated individuals will still get infected [7], despite that; it is expected that those individuals will experience a less severe form of the infection, less frequent hospitalization, and intensive care unit (ICU) admission [[7], [8], [9]], this is possibly achieved through the vaccines' ability to produce immunological memory responses, that hasten the removal of infected cells, while reducing viral replication [10]. Indeed, several studies that examined post-vaccination COVID-19 cases support such an expectation. In a case control study, the vaccine's effectiveness after the second dose in preventing hospitalization was 87% [11], while a prospective cohort study concluded that COVID-19 vaccinated cases will suffer lower duration of illness, and risk of febrile symptoms [9].

Most of the studies on post-vaccination hospitalized COVID-19 cases evaluate their severity by ICU admission, mechanical ventilation, and mortality [7,12,13]. To our best knowledge, no study has attempted to objectively determine the severity of COVID-19 infection among vaccinated patients admitted to ICU, hence; this study was carried out under the hypothesis that among COVID-19 patients admitted to ICU, vaccinated patients may have a lower disease severity compared to unvaccinated.

## 2 Methods

This was a prospective observational cohort study, performed at the ICU of King Saud Medical City (KSMC). KSMC is the largest government hospital in the central region of Saudi Arabia, harboring 1200 beds. The ICU originally included 127 beds, and was expanded to accommodate the surge in COVID-19 patients during the second wave (as of January 2021), to include 300 beds. It is a closed ICU, operated by intensivists round the clock, with a 1:1 Nurse to patient ratio. Since the beginning of the COVID-19 era, KSMC has been a COVID-19 referral center, generally following COVID-19 management recommendations issued by the Ministry of Health [14]. The study was approved by the local institutional review board (Reference number: H1R1-22-June 21-04) with waiver of consent in view of its observational design, and has been registered in Research Registry under UIN number: researchregistry7222.

*Patients and timeframe:* All hospitalized COVID-19 patients between January 1st, 2021 and June 30th, 2021 were eligible for inclusion if they fulfilled the following criteria: Adult (Age ≥18 years), confirmed COVID-19 diagnosis by Reverse transcription polymerase chain reaction (RT-PCR) of nasopharyngeal swabs, and admitted to ICU. We excluded pregnant ladies, patients known to have pulmonary tuberculosis (PTB), and Human immunodeficiency virus (HIV) positive patients.

*Data management:* For all enrolled patients during the study period we collected baseline demographic and chronic health conditions (age, gender, nationality, and comorbidities). Furthermore, we noted the clinical characteristics upon admission to the hospital, this included vital signs, peripheral oxygen saturation on room air (SpO_2_), supplemental oxygen requirement (provided at the discretion of the treating physician to maintain SpO_2_ ≥ 94%), and respiratory rate. This enabled categorization of patients based on severity upon hospital admission according to an ordinal scale [15]. Enrolled patients were followed for their period of hospital stay.

Initially, we planned to group patients according to immunization status into 3 groups (No immunization, single dose, and two doses), however; by the conclusion of the study period, we have enrolled only five patients with two doses of immunization, accordingly; we divided the patients into two groups of either no immunization, or at least one dose to avoid disadvantages of a small sized group in the statistical analysis [16]. The manufacturer of the vaccine was not taken into consideration (COVID-19 vaccines approved in Saudi Arabia at the time of the study are Pfizer ® and Astra-Zineca ®), we also recorded the duration between the last dose of vaccination (for vaccinated patients) and first development of symptoms. Treatment variables were recorded in terms of general medications groups, to include: Tocilizumab, steroids, and antivirals. Finally, each patient's 30 day outcome was recorded as a binary dead or alive variable, along with hospital and ICU length of stay (LOS). Patients transferred to other hospitals were not followed there, and were considered as alive and discharged from our hospital.

*Outcomes:* The primary outcome of the study was to compare severity of admission among COVID-19 patients admitted to ICU according to immunization status. Secondary outcomes included, 30 day all-cause mortality, ICU and hospital LOS, and requirement of mechanical ventilation upon ICU admission.

### 2.1 Statistical analysis

Continuous variables were summarized as mean ± standard deviation (SD) and median interquartile range (IQR), and were compared between groups by student *t*-test or Mann Whitney *U* test as appropriate without normalization, discrete variables were summarized as frequency and percentage (%), and were compared between groups by chi square test or Fisher's exact test as appropriate, without correction for multiple testing. Test results were presented with corresponding 95% confidence interval (CI) of difference, and p values.

The primary outcome of severity ordinal scale was evaluated using proportional odds model for ordinal outcomes, presenting results as odds ratio (OR) adjusted for age and gender, likelihood ratio test was used to evaluate the parallel regression assumption (assumption holds if p > 0.05). Whereas the association of immunization status with 30 day mortality was explored in a logistic regression model, initially, all significant variables were identified in a univariable model, and the final multivariable model included variables with p < 0.1 in the univariable model, with evaluation of fulfilment of the assumptions of logistic regression and goodness of fit (details in supplementary file).

All statistical tests were two tailed, and considered significant with p values < 0.05. Statistical tests were carried out by commercially available software: STATA® [StataCorp. 2019. *Stata Statistical Software: Release 16*. College Station, TX: StataCorp LLC.].

This research has been reported in line with the STROCSS criteria [17].

## 3 Results

**Fig. 1:**
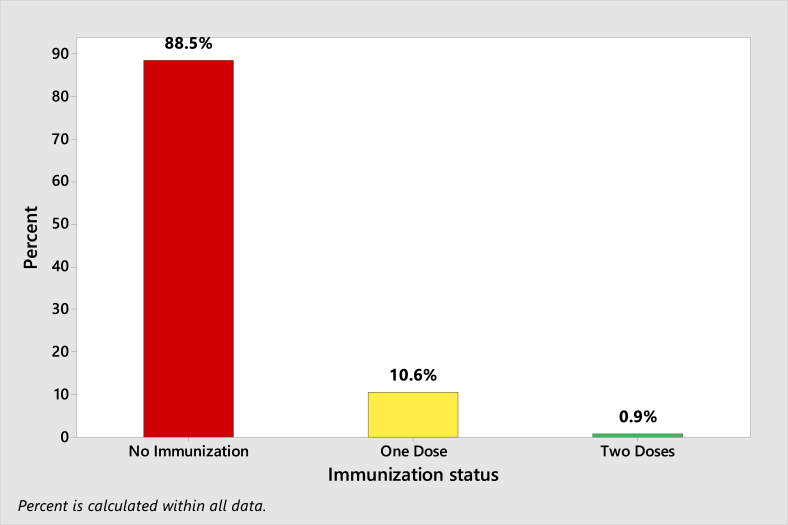
Percentage of enrolled patients by immunization status.

During the study period there were 611 admissions to ICU with confirmed COVID-19 diagnosis, seven pregnant ladies, and 12 minor patients were excluded, leaving 592 patients to be enrolled. 524 (88.5%) patients have not received any immunization, 63 (10.6%) patients received a single dose, and only five patients (0.9%) received two doses of immunization (Fig. 1), the average duration between the last immunization dose and first appearance of symptoms was 13.5 ± 6.7 days (for 68 patients, Fig. S1), with majority of patients (86.8%) showing symptoms within 21 days of vaccination (Fig. S2), notably, all five patients with two doses of immunization showed symptoms after more than 21 days of the second dose. Due to our prospective follow up of enrolled patients, there were no missing data. The patients were grouped into two groups of No immunization (thereafter called group 1) and at least one dose of immunization (thereafter called group 2). Demographic and clinical characteristics of both groups were similar with the exception of a higher percentage of patients in group 2 with three or more comorbidities (Table 1), the similarity of both groups with regards to demographic and management modalities may be an indication that medications received in ICU may not be a confounding factors.

**Table 1:** Demographic, clinical characteristics, and outcomes.

Variable	Group 1 (n = 524)	Group 2 (n = 68)	95% CI of difference	P value
Mean Age (years)				0.13*
Mean ± SD	54 ± 16.1	57.1 ± 12.5	−7.1 to 0.9	
Median (IQR)	54 (44–65)	58 (49.5–65)		
Gender (Male)	357 (68.1%)	54 (79.4%)	−3.2%–10.5%	0.06
Number of Comorbidities:				
No	267 (50.9%)	34 (50%)	−12.2%–14%	0.99
One	109 (20.8%)	7 (10.3%)	−0.2%–17.6%	0.06
Two	112 (21.4%)	17 (25%)	−6.8%–16.1%	0.6
Three or more	36 (6.9%)	10 (14.7%)	0.04%–18.7%	0.04
Main Comorbidities:				
DM	179 (34.2%)	26 (38.2%)	−8.3%–17.2%	0.5
HTN	166 (31.7%)	28 (41.2%)	−3%–22.7%	0.1
Asthma or COPD	26 (5%)	2 (2.9%)	−5.4%–5.5%	0.5
CKD	27 (5.2%)	8 (11.8%)	−0.3%–16.9%	0.06
IHD	22 (4.2%)	3 (4.4%)	−3.9%–8.3%	0.9
HF or Arrhythmia	9 (1.7%)	3 (4.4%)	−1.1%–10.7%	0.1
Malignancy	8 (1.5%)	1 (1.5%)	−2.1%–6.5%	0.9
Ischemic Stroke	13 (2.5%)	2 (2.9%)	−2.7%–7.8%	0.8
Liver Disease	4 (0.8%)	1 (1.5%)	−1.2%–7.2%	0.5
Ordinal scale of severity upon hospital admission:				
Category 3	15 (2.9%)	11 (16.2%)	5.3%–24.3%	<0.001
Category 4	145 (27.7%)	31 (45.6)	5.11%–31%	0.004
Category 5	222 (42.4%)	16 (23.5%)	6.3%–29.3%	0.004
Category 6	142 (27.1%)	10 (14.7%)	1.1%–20.8%	0.04
MV upon ICU Admission	156 (29.8%)	15 (22.1%)	−4.6%–17.8%	0.2
Patients Initially Admitted to Ward	181 (34.5%)	17 (25%)	−3.2%–20.1%	0.2
Medications In ICU:				
Tocilizumab	37 (7.1%)	6 (8.8%)	−4.4%–11.3%	0.8
Steroids	149 (28.8%)	18 (26.5%)	−10.4%–13.1%	0.8
Antiviral	179 (34.2%)	21 (30.1%)	−8.9%–15.5%	0.6
30-day Mortality	230 (43.9%)	20 (29.4%)	1.5%–25.8%	0.02
ICU LOS:				
Mean	15.4 ± 10.8	15.8 ± 9.5	−3.1 to 2.3	0.4**
Median	13 (8–20)	13 (9–21)		
Hospital LOS:				
Mean	18.5 ± 11	19.1 ± 10.1	−3.4 to 2.1	0.4**
Median	16 (11–23)	16.5 (12.5–24)		

*Student t-test, **Mann Whitney *U* test, otherwise Chi square test.

SD = standard deviation, IQR = interquartile range, DM = diabetes mellitus, HTN = hypertension, COPD = chronic obstructive pulmonary disease, CKD = chronic kidney disease, IHD = ischemic heart disease, HF = heart failure, MV = mechanical ventilation, LOS = length of stay, ICU = intensive care unit.

**Table 2:** Ordinal regression of severity scale.

Variable	OR	95% CI	P value
Immunization status	0.2	0.15 to 0.4	<0.001
Age	1.01	1.003 to 1.02	0.01
Gender	0.7	0.5 to 0.9	0.012

*Primary outcome*: Table 1 and Fig. S3 show the distribution of the ordinal scale of severity upon admission across the two groups. Group 1 had significantly lower percentages than group 2 in the lower two categories (categories 3 and 4), while higher percentages were observed in the higher two categories (categories 5 and 6). Immunization status (adjusted for age and gender) was significantly associated with lowering the odds of moving up the ordinal scale of severity (OR 0.2 [95% CI: 0.15 to 0.4]; p < 0.001), likelihood ratio test was not statistically significant (p = 0.3) indicating fulfillment of the parallel regression assumption (Tables 2 and S2).

*Secondary outcomes*: The percentage of patients requiring MV upon ICU admission was higher in group 1 than group 2, however, the difference didn't reach statistical significance (26.9% Vs 19.1%, 95% CI: −4.2%–17.2%; p = 0.2). In group 1 all-cause 30 day mortality was 230/524 (43.9%), while that for group 2 was 20/68 (29.4%), the difference was statistically significant (95% CI: 1.5 to 25.8; p = 0.02). On the contrary, neither ICU nor hospital LOS was different between both groups (Table 1).

Several variables were identified to be associated with 30 day mortality in univariable logistic regression, including: Age, immunization status, number of comorbidities, severity of admission, MV on admission, diabetes mellitus, hypertension, chronic kidney disease, coronary heart disease, and heart failure or arrhythmia. When all the variables were evaluated in a multivariable logistic regression model, immunization status was significantly associated with reduction of the odds of 30 day mortality (OR 0.45 [95% CI: 0.23 to 0.89]; p = 0.02) (Table 3). The model was well fitted (Hosmer-Lemeshow p value = 0.9), with fulfillment of assumptions of logistic regression (Table S3 – S5) and a statistically significant area under the curve for the model (AUC = 0.788 [95% CI: 0.753–0.82]; p < 0.001). (Fig. S4).

**Table 3:** Logistic regression of 30 day mortality.

Variable	Univariable Model			Multivariable Model		
	OR	95% CI	P value	OR	95% CI	P value
Age	1.04	1.03–1.05	<0.001	1.03	1.02–1.04	<0.001
Gender	1.02	0.7–1.5	0.9	–	–	–
Immunization status	0.5	0.3–0.9	0.02	0.5	0.2–0.9	0.02
Number of comorbidities	1.8	1.5–2.1	<0.001	3.19	1.6–6.4	0.001
Diabetes Mellitus	2	1.4–2.8	<0.001	1.4	1.01–1.7	0.047
Hypertension	2.1	1.5–2.9	<0.001	1.3	1.1–1.5	0.012
Asthma or COPD	1.6	0.8–3.5	0.2	–	–	–
CKD	6	2.6–14.1	<0.001	1.9	0.6–6	0.3
CHD	2.1	0.9–4.8	0.07	1.3	1.02–1.7	0.04
Heart failure/Arrhythmia	15.7	2–122.4	0.009	3.6	0.4–31.9	0.2
Malignancy	1	–	–	–	–	–
CVA	1	–	–	–	–	–
Liver Disease	5.5	0.6–50	0.13	–	–	–
Admission Severity	2.2	1.8–2.7	<0.001	1.4	1–2	0.05
MV on Admission	4.5	3.1–6.6	<0.001	2.7	1.5–4.9	0.001

COPD = chronic obstructive pulmonary disease, CKD = chronic kidney disease, CHD = coronary heart disease, CVA = cerebrovascular accident, MV = mechanical ventilation.

## 4 Discussion

In this study we examined the data of 592 COVID-19 positive patients admitted to the ICU during the study period, there was an obvious predominance of unvaccinated patients in the cohort, with only about 11% who had received at least one dose of vaccination. This is not surprising as both vaccine brands authorized in Saudi Arabia have already demonstrated excellent efficacy in RCTs [4,5]. Despite the fact that the majority of the vaccinated group were partially immunized, being a minority in the whole cohort is comprehendible, as several studies demonstrated vaccine efficacy between 80% and 91% after the first dose [18,19]. The immunized group slightly differed from the non-immunized by including significantly more patients with three or more comorbidities, which places them at a higher risk of COVID-19 infection compared to others [11,20]. More importantly, most of the patients who had received at least one dose of vaccination presented with symptoms and were hospitalized within less than 21 days of their vaccination as immunity develops over time. During the first two to three weeks after the first dose of vaccination efficacy is much lower than afterwards [7], as spike immunoglobulins G (IgG) begin to appear around this period [13]. Infection of partially immunized patients within 2–3 weeks of immunization - rather than afterwards - is a common finding in many studies on post vaccination COVID-19 cases [13,21]. In our cohort there were five patients who were fully immunized, they presented after 21 days had passed since their last immunization dose, leading to the assumption that those patients were non-responders (sometime referred to as breakthrough infections). Since most COVID-19 vaccines are based on the development of spike IgG antibodies, their efficacy may be attenuated by antigen mutation [12], and it was during the study period that new variants – possibly with antigen mutation – started to appear worldwide, and possibly in Saudi Arabia [22].

The majority of clinical trials on post-vaccination COVID-19 addressed severity of the disease in terms of either symptomatic or asymptomatic presentation, or count of events of hospitalization, ICU admission, mechanical ventilation, or death [7,9,11,13,23,24], unanimously concluding lower severity (according to the design of each study) in the group of immunized patients. This was also true for our secondary outcome of all-cause 30 day mortality, which was significantly lower in the immunized group. However, for our primary outcome, we chose to be more thorough regarding categorization of severity upon hospital admission utilizing an ordinal scale of severity that was previously applied to COVID-19 patients [15]. And by applying ordinal regression, we demonstrated that immunization by at least one dose of vaccination is not only associated with lower severity upon hospitalization, but it uniformly lowers the odds of moving from one category to the next, that is to say, the effect of the independent variable (at least one dose of immunization in this case) has the same effect on any pair of consecutive outcomes of the ordinal scale [25]. Accordingly, our model – which we believe is unprecedented - indicates that at least one dose of immunization decreases the odds of changing from severity category 3 to 4, 4 to 5, and 5 to 6 by 80%. The mechanism, however; by which partial immunization attenuates the disease remains mostly unknown, with hypotheses based on other types of vaccines suggesting reduction of viral replication, and rapid elimination of infected cells through recall of immunologic memory responses [10,26]. In support of this finding, was the results of well fitted multivariable logistic regression model showing that at least one dose of immunization lowers the adjusted odds of 30 day all-cause mortality by 55%.

Our study suffers numerous limitations, this was an observational study lacking the advantages of randomization due to obvious ethical reasons, however; statistical methods could be used in future studies such as propensity score matching or emulation studies to produce more valid results. The sample size is relatively small and may have affected the power of the study, although this was possibly compensated for by rigorous statistical model. This was a single center study, reflecting the management of only one hospital. Also data on variants and impact on disease severity is lacking. And finally, our analysis lacks subdivision of immunized patients by duration since vaccination, which could have produced more informative results.

## 5 Conclusion

In our study the majority of COVID-19 patients admitted to ICU were non-immunized, most of the partially immunized patients got infected before immunity could develop, and the minute percentage of fully immunized patients probably were non-responders. Receiving at least one dose of immunization significantly decreases severity of the disease across all ordinal severity categories. Furthermore, at least one dose of immunization is significantly associated with lower 30 day all-cause mortality. Policy makers and clinicians may need to take the immunization status in consideration when deciding on disposition of COVID-19 patients to ICU or general ward.

## Ethical approval

The study was approved by the local institutional review board (Reference number: H1R1-22-June 21-04) with waiver of consent in view of its observational design.

## Funding statement

No external funding was received for this work.

## Author contribution

All authors contributed equally.

## Ethical approval

King Saud Medical City IRB approval obtained (Reference number: H1R1-22-June 21-04).

## Consent

IRB approval with waiver of consent in view of its observational design.

## Registration of research studies

1. Name of the registry: Research Registry
2. Unique Identifying number or registration ID: 7162
3. Hyperlink to your specific registration (must be publicly accessible and will be checked): https://www.researchregistry.com/browse-the-registry#home/

## Guarantor

Prof. Ziad A Memish.

## Declaration of competing interest

All authors declare no conflict of interests.
