# Supplementary material for: Comparison of severity of immunized versus non-immunized COVID-19 patients admitted to ICU: A prospective observational study
Source: Ann Med Surg (Lond). 2021 Oct 15;71:102951. doi: 10.1016/j.amsu.2021.102951 (PMC8518130; doi:10.1016/j.amsu.2021.102951)
Supplement: Multimedia component 2 [file mmc2.docx]

**Supplementary file:**

Table S1: Severity 7-level ordinal scale:

| Clinical status: | Severity score |
| --- | --- |
| Not admitted to hospital, no limitation of activities | 1 |
| Not admitted to hospital, with limitations of activities | 2 |
| Admitted to hospital, not receiving supplemental oxygen | 3 |
| Admitted to hospital, receiving supplemental oxygen (nasal cannula, face mask, non-rebreathing face mask) of any flow. | 4 |
| Admitted to hospital, receiving non-invasive mechanical ventilation (CPAP, BiPAP) or Oxygen via high flow nasal cannula | 5 |
| Admitted to hospital, mechanically ventilated. | 6 |
| Dead | 7 |

From:

Veiga VC, Prats JAGG, Farias DLC, et al. Effect of tocilizumab on clinical outcomes at 15 days in patients with severe or critical coronavirus disease 2019: randomised controlled trial. BMJ. 2021;372:n84.

Table S2: Approximate likelihood ratio test of proportionality of odds across response categories:

Chi square p value > 0.05

Parallel regression assumption is fulfilled.

Table S3: Variable inflation factors of multivariable logistic regression model:

| Variable | VIF | 1/VIF |
| --- | --- | --- |
| Hypertension | 3.95 | 0.25 |
| Diabetes Mellitus | 3.61 | 0.28 |
| Number of comorbidities | 2.9 | 0.34 |
| Severity of Admission | 2.07 | 0.48 |
| Chronic kidney disease | 1.5 | 0.67 |
| Coronary heart disease | 1.31 | 0.76 |
| Age | 1.18 | 0.85 |
| Heart failure / Arrhythmia | 1.17 | 0.85 |
| Immunization status | 1.09 | 0.92 |
| Mean VIF | 2.09 |  |

All VIF < 4

Table S4: Correlation matrix of continuous variables in multivariable logistic regression model:

|  | Age | Number of comorbidities | Severity scale |
| --- | --- | --- | --- |
| Age |  | 0.3 | 0.1 |
| Number of comorbidities | 0.3 |  | 0.1 |
| Severity scale | 0.1 | 0.1 |  |

Weak correlation of continuous variables in the model, fulfills the assumption of absent multi-collinearity.

Table S5: Linearity between continuous variables in the logistic regression model and log transformation of dependent variable (Box-Tidwell test):

| Age | .0324164 | .0068396 | 4.74 | Nonlin. | dev. | 3.517 | (P | = | 0.061) |
| --- | --- | --- | --- | --- | --- | --- | --- | --- | --- |
| p1 | 5.264041 | 2.034719 |  |  |  |  |  |  |  |
| Number of comorbidities | 1.07895 | .3565988 | 3.03 | Nonlin. | dev. | 0.678 | (P | = | 0.410) |
| p1 | .5219587 | .6407234 |  |  |  |  |  |  |  |
| Severity of Admission | .4210843 | .17615 | 2.39 | Nonlin. | dev. | 0.060 | (P | = | 0.807) |
| p1 | .4430589 | 4.729442 |  |  |  |  |  |  |  |
|  |  |  |  |  |  |  |  |  |  |

All p values > 0.05

Assumption of linearity of continuous variables and Log transformation of dependent variable is fulfilled.

Figure S1: Time to development of symptoms from last dose of immunization:

Graph for patients with at least one dose of immunization (n = 68)

Mean ± SD: 13.5 ± 6.7

Median (IQR): 13 (8.5 – 17)

Figure S2: Vaccination to symptoms categorization:

95% CI of difference: 58.9% to 83.5%

P < 0.001

Figure S3: Severity Ordinal Scale across Groups:

Figure S4: Area under the curve of logistic regression prediction model of 30 day mortality:

AUC = 0.788 (95% CI: 0.753 – 0.82)
